# High charge mobility in two-dimensional percolative networks of PbSe quantum dots connected by atomic bonds

**DOI:** 10.1038/ncomms9195

**Published:** 2015-09-24

**Authors:** Wiel H. Evers, Juleon M. Schins, Michiel Aerts, Aditya Kulkarni, Pierre Capiod, Maxime Berthe, Bruno Grandidier, Christophe Delerue, Herre S. J. van der Zant, Carlo van Overbeek, Joep L. Peters, Daniel Vanmaekelbergh, Laurens D. A. Siebbeles

**Affiliations:** 1 Optoelectronic Materials Section, Department of Chemical Engineering, Delft University of Technology, Julianalaan 136, 2628 BL Delft, The Netherlands; 2 Kavli Institute of Nanoscience, Delft University of Technology, 2628 CJ Delft, The Netherlands; 3 Institut d'Electronique, de Microélectronique et de Nanotechnologie (IEMN), CNRS, UMR 8520, Département ISEN, 41 bd Vauban, 59046 Lille Cedex, France; 4 Debye Institute for Nanomaterials Science, University of Utrecht, Princetonplein 1, 3584 CC Utrecht, The Netherlands

## Abstract

Two-dimensional networks of quantum dots connected by atomic bonds have an electronic structure that is distinct from that of arrays of quantum dots coupled by ligand molecules. We prepared atomically coherent two-dimensional percolative networks of PbSe quantum dots connected via atomic bonds. Here, we show that photoexcitation leads to generation of free charges that eventually decay via trapping. The charge mobility probed with an AC electric field increases with frequency from 150±15 cm^2^ V^−1^ s^−1^ at 0.2 terahertz to 260±15 cm^2^ V^−1^ s^−1^ at 0.6 terahertz. Gated four-probe measurements yield a DC electron mobility of 13±2 cm^2^ V^−1^ s^−1^. The terahertz mobilities are much higher than for arrays of quantum dots coupled via surface ligands and are similar to the highest DC mobilities reported for PbSe nanowires. The terahertz mobility increases only slightly with temperature in the range of 15–290 K. The extent of straight segments in the two-dimensional percolative networks limits the mobility, rather than charge scattering by phonons.

## Abstract (web summary)

The effect of nanocrystal structure on electronic properties is of considerable interest for optoelectronic devices. Here, Evers *et al*. study the charge transport in two-dimensional percolative networks of PbSe and find excellent terahertz mobility of charge carriers.

## Graphical abstract

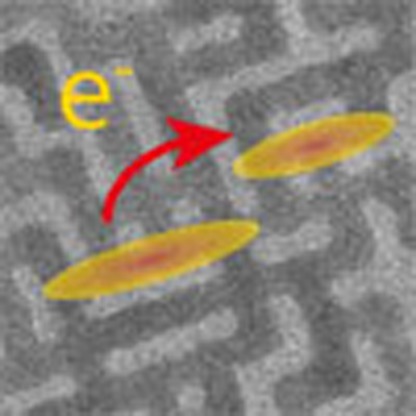

Colloidal semiconductor nanocrystals are of interest because their electronic and optical properties can be tuned by variation of composition and shape. The effects of nanocrystal structure on electronic properties are of fundamental scientific interest and offer challenging prospects for application in (opto-)electronic devices such as transistors, light-emitting diodes, photovoltaic cells, lasers, thermoelectric modules and sensors^1^,^2^,^3^. Colloidal nanocrystals have been prepared as quantum dots (QDs)^1^, nanorods^2^ and continuous two-dimensional (2D) nanosheets, also known as colloidal quantum wells^3^,^4^. Very recently, 2D square and honeycomb networks of colloidal lead- and cadmium-chalcogenide QDs have been prepared^5^,^6^. These networks are formed by oriented attachment of QDs, which are fused by bonds between cations and anions on opposite facets. The necks connecting the QDs have a thickness close to the QD diameter, which is much larger than realized after displacement or partial removal of surface ligands in PbSe QD films, as reported in refs 7 and 8. Theoretical studies have shown that the necking of QD facets causes the electronic band structure of the 2D networks to be profoundly distinct from continuous nanosheets, with the appearance of Dirac cones and non-trivial flat bands in the case of a honeycomb network^9^,^10^,^11^.

Strong inter-dot electronic coupling via atomic necking in a network may result in an even higher charge mobility than the values of a few up to tens of cm^2^ V^−1^ s^−1^, reported for arrays of self-assembled lead chalcogenide QDs that are less strongly coupled by small (in)organic surface ligand molecules^12^,^13^,^14^,^15^,^16^, or necks of atomic bonds with diameter significantly less than that of the original QDs^7^,^8^. To exploit the atomic necking in a device configuration, the length of uninterrupted segments of attached QDs should be as long as possible and preferentially approach the inter-electrode distance. A higher mobility, while maintaining the nanostructuring and effects of quantum confinement, would be an asset in optoelectronic devices such as mid- and near-infrared photodetectors and solar cells, and also for thermoelectric devices for which Pb-chalcogenides are favourable material systems. A cheap wet-chemical ‘bottom-up' preparation of thin-film networks is of high advantage for large-scale applications as compared with expensive lithographic ‘top-down' approaches. It is hence of large importance to obtain reliable values of the charge carrier mobility and to study the factors that govern charge transport in these nanostructured but single-crystalline networks.

Here, we report the photogeneration, mobility and decay of charge carriers in 2D percolative networks of PbSe. Steady-state electrical transport that is intimately related to the degree of connection along the charge carrier pathways reveals DC electron mobilities up to 13±2 cm^2^ V^−1^ s^−1^. Terahertz (THz)-frequency conductivity measurements, that probe charge carrier motion for typical lengths corresponding to several bonded QDs, show an increase of the mobility with frequency of the probing electric field. In the THz frequency range, the mobility is hardly dependent on temperature, which implies that the length of the segments in the percolation network limits the mobility, rather than charge scattering by phonons. Values as high as 260±15 cm^2^ V^−1^ s^−1^ have been observed, not too far from the room temperature PbSe bulk value of 1,000 cm^2^ V^−1^ s^−1^ (refs 17, 18, 19). This suggests that improving long-range order in the 2D sheets could further enhance the DC charge mobility, offering tremendous prospects for the abovementioned device applications.

## Results

### Preparation and characterization of 2D PbSe networks

**Figure 1:**
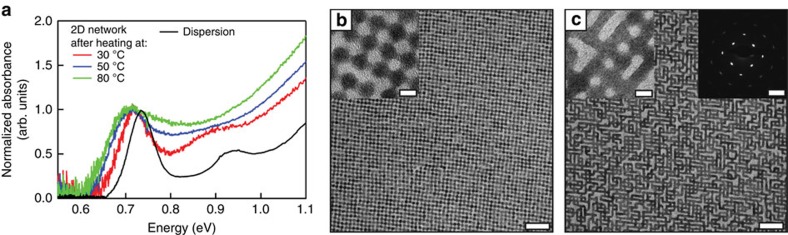
Optical absorption spectra and structure of 2D PbSe networks. (**a**) Optical absorption spectra of 2D PbSe networks and PbSe QDs in dispersion. (**b**) TEM image of a network of relatively weakly coupled QDs obtained by heating at 30 °C (15 min). (**c**) 2D percolative network of fused QDs obtained after subsequent heating in two steps, first at 50 °C (15 min) and subsequently at 80 °C (15 min). The presence of diffraction spots in the electrodiffractogram in the upper right corner implies that crystal planes in different QDs have the same orientation. The scale bars for the TEM images, TEM insets and electrodiffractogram represent 40 nm, 5 nm and 0.25 nm, respectively.

We prepared 2D percolative networks with thickness of one nanocrystal by oriented attachment of PbSe QDs with diameter 5.8±0.4 nm (see Methods), according to the procedure published previously^5^,^6^. The optical absorption spectrum of the QDs in tetrachloroethylene dispersion exhibits a peak at 0.73 eV due to the first exciton transition, see Fig. 1a. The 2D networks are prepared by first placing a dispersion of QDs in toluene on top of a volume of ethylene glycol (EG), containing oleic acid in a glass vial under nitrogen atmosphere at 25 °C. Since toluene and EG do not mix a two-phase system is formed. Subsequently, the system is left for 1 h, during which the toluene evaporates and the initial atomically coherent (pseudohexagonal) monolayer of oriented QDs is formed (see Supplementary Fig. 1). Heating at 30 °C for 15 min leads to limited atomic necking of the QDs and formation of a 2D superstructure with square geometry, see the transmission electron microscope (TEM) image in Fig. 1b. The thickness of the necks between the QDs is still significantly less than their diameter. The absorption peak at low-photon energy of this superstructure is red-shifted and broadened as compared with that for the QD dispersion. These effects are due to change of the dielectric environment^20^ and the occurrence of electronic coupling between QDs^21^ on going from the dispersion to the superstructure. Further heating at 50 °C for 15 min and 80 °C for 15 min leads to fusion of QDs by necks of thickness 4.0±0.4 nm, which is close to the diameter of the original QDs, see Fig. 1c. Necking causes the centre-to-centre distance between the fused QDs to become 6.4±0.1 nm. The average number of necks per QD is 2.6±0.7, which implies that the system is a 2D percolative network. The percolative network contains straight segments with an average length of 20 nm, corresponding to 3–4 QDs. The gap between QDs that are not connected by a neck is 2.3±0.4 nm. The necking causes further broadening of the optical absorption peak, see Fig. 1a. An electron diffractogram measured over an area of several square microns is shown in the top right corner of Fig. 1c. The presence of diffraction spots, rather than rings, implies that the crystal planes of different QDs have the same orientation.

The laser spectroscopic studies described below were carried out on stacks of six layers of 2D percolative PbSe networks with a structure as shown in Fig. 1c, obtained after heating at 30, 50 and 80 °C as described above. The optical absorption spectrum of the sample is shown in Supplementary Fig. 2. As described in the Methods section, and shown in Supplementary Fig. 3, the laser spectroscopic results for a single layer and a stack of layers are identical. Hence, there are no effects of fusion between the stacked layers. The THz conductivity of non-annealed samples and those annealed only at 30 °C was below the noise level, implying a mobility below 10 cm^2^ V^−1^ s^−1^. From this, we infer that charge transfer over a gap between QDs that are not connected by a neck is negligible. In what follows, we only consider samples annealed at 80 °C as described above.

### Photogeneration and decay of charge carriers

Below we discuss our studies on the 2D percolative networks (Fig. 1c), obtained after heating at 30, 50 and 80 °C, as specified above. The optical absorption spectrum of the sample is shown in Supplementary Fig. 2. In all experiments on photogeneration, THz mobility and decay of charge carriers, the sample was excited with 60 fs pump laser pulses (see Methods). The data discussed below were obtained using an absorbed pump fluence of 1.4 × 10^12^ photons per cm^2^ or less (≤0.1 excitations per QD volume), which was found to be sufficiently low so that Coulomb interactions and higher-order recombination do not affect the mobility and decay kinetics of the charges, see Supplementary Fig. 4.

**Figure 2:**
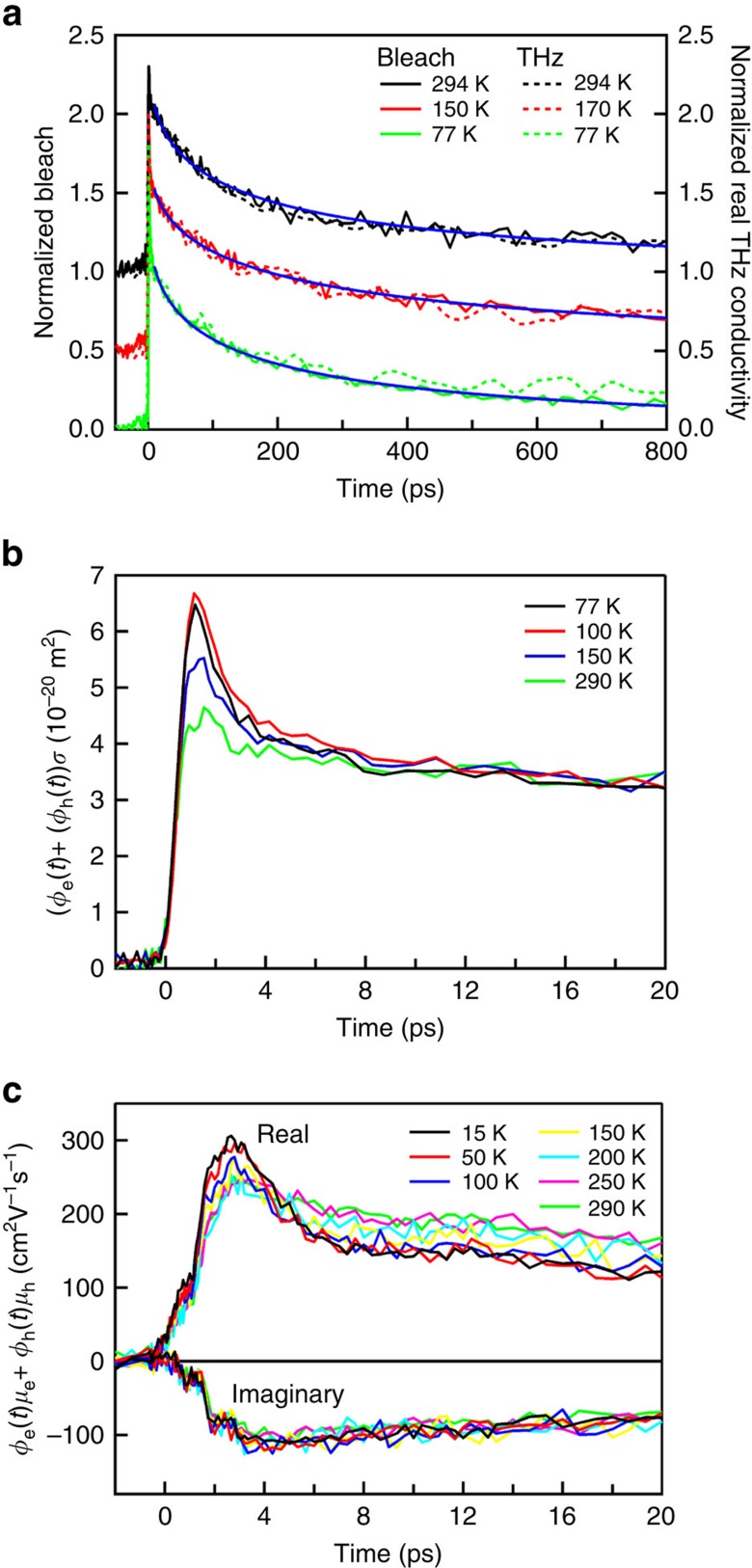
Optical bleach and THz conductivity after photoexcitation. (**a**) Long timescale optical bleach (left axis) and real component of the THz conductivity (right axis) normalized at a time of 10 ps and offset vertically for clarity. The dashed curves are fits of a stretched-exponential. (**b**) Short timescale product of the sum of the quantum yields of electrons and holes, *ϕ*_*e*_(*t*)+*ϕ*_*h*_(*t*), and the cross section for bleach, *σ* (see Methods). (**c**) Short timescale sum of the quantum yield of electrons and holes, weighted by their real or imaginary mobility, *μ*, averaged over the frequency range 0.3–0.5 THz (see Methods).

**Figure 3:**
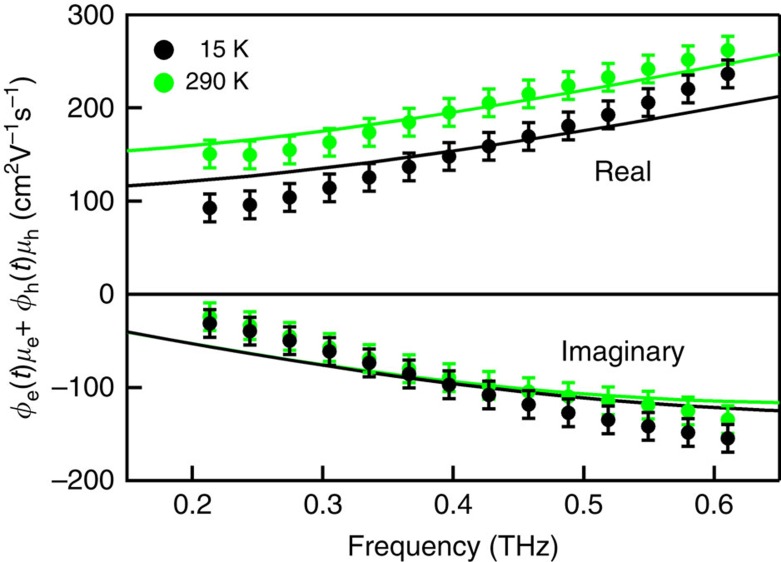
Frequency dependence of the charge mobility. Real (upper panel) and imaginary (lower panel) components of the charge mobility at 15 and 290 K, averaged over pump–probe time delays in the interval 8–12 ps. The uncertainty in the mobility data is determined by fluctuations in the pump laser fluence. The increase of the magnitude of the real and imaginary mobility is typical for charge motion that is hindered by scattering at barriers to transport. The solid curves are fits of the Drude-Smith model to the experimental mobility data.

Figure 2a shows the normalized optical bleach at a photon energy of 0.70 eV due to electrons and holes at the band edge, together with the normalized THz conductivity, obtained on photoexcitation at 1.55 eV (800 nm) at temperatures as indicated. Optical bleach and THz conductivity data on a shorter timescale are shown in Fig. 2b,c. The electrons and holes can be coulombically bound in the form of excitons, or behave as free charges. As discussed in Section 3 of the Supplementary Information, the frequency dependence of the measured THz conductivity (Fig. 3; Supplementary Figs 6 and 7) precludes excitons or plasmons as its origin. This leads to the conclusion that the THz conductivity is due to free electrons and holes that move independently. The similar decay kinetics of the bleach and real component of the THz conductivity in Fig. 2a exclude the existence of an independent population of excitons. The formation of free charges was also inferred from previous THz photoconductivity studies on films of PbSe QDs connected by molecular surface ligands^12^,^22^.

Effects due to initially hot charge carriers resulting from photoexcitation above the band gap do not play a role, since results obtained for photoexcitation at 800 nm are similar to those for photoexcitation at the band edge (1,700 nm), see Supplementary Fig. 5. The bleach and THz conductivity decay on a timescale of hundreds of picoseconds, which is more than three orders of magnitude shorter than the 800 ns lifetime for recombination of an electron-hole pair in a PbSe QD in dispersion^23^,^24^. Therefore, we attribute the decay of the data in Fig. 2 to trapping of charges at localized states with energy in the band gap.

During the first few picoseconds the bleach in Fig. 2b and the real THz conductivity in Fig. 2c exhibit a fast-decaying component with a magnitude that decreases as the temperature goes up. This is attributed to ultrafast thermally activated trapping of either electrons or holes. The ultrashort-lived charges do not give rise to a fast-decaying component of the imaginary THz conductivity. The negligible imaginary mobility of these charges implies that they move with a drift velocity in-phase with the THz electric field without being hindered by boundaries or barriers that impede their motion^25^,^26^,^27^.

The optical bleach and THz conductivity in Fig. 2 on times exceeding the first few picoseconds is due to either electrons or holes that survive before trapping on a longer timescale of the order of 100 ps. The product of the quantum yield and the real THz mobility of the long-lived charges in Fig. 2c exhibit a slight increase with temperature, while the imaginary mobility is independent of temperature. It is to be expected that the diffusion coefficient, *D*, of charges increases with temperature according to the Einstein relation *D*=*μ*_*DC*_*k*_*B*_*T*/*e*, with *μ*_*DC*_ the DC charge mobility, *T* the temperature, *k*_*B*_ the Boltzmann constant and *e* the elementary charge. If charge trapping would be diffusion controlled, the decay of the charges on longer times should be faster at higher temperature in contrast to the data in Fig. 2. Hence, the decay time of charges is not determined by thermally activated diffusion to trapping sites. It is inferred that charges are continuously in contact with trapping sites and that the decay time of the order of 100 ps is determined by the trapping rates. The trapping kinetics can be described by a stretched-exponential function of the form 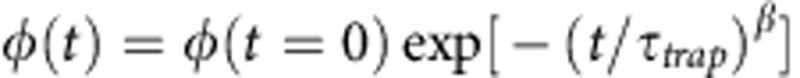, with temperature-independent lifetime *τ*_*trap*_=115±45 ps and stretch-parameter *β*=0.4±0.1, see Fig. 2a. Such stretched-exponential decay is typical for a distribution of trapping rates^28^.

### Charge mobility

The frequency dependence of the mobility of the long-lived charges was obtained from averaging THz photoconductivity data as in Fig. 2c over a pump–probe delay interval of 8–12 ps, at which the short-lived charges have decayed. The quantum yield of the long-lived charges in this time interval was taken equal to the maximum value of 1. This, together with partial neglect of effective medium effects leads to mobility values that can be somewhat lower than the actual values, as discussed in Methods and Section 3 of the Supplementary Information. Results for temperatures of 15 and 290 K are shown in Fig. 3. Mobility data at intermediate temperatures are presented in Supplementary Fig. 6. The results in Fig. 3 show that the real component of the mobility increases with frequency, which is typical for charges moving between barriers to transport^26^,^29^,^30^. The real mobility determines the velocity of a charge in-phase with the oscillating THz field, see Methods. As the frequency increases, it becomes less likely that a charge encounters a barrier during an oscillation period of the THz field. Hence, at higher frequency the charges can better follow the THz field by moving forward and backward without being hindered by barriers. In this way, the phase-lag between the charge velocity and the THz field becomes smaller and the real mobility increases with frequency. The real mobility at the THz frequencies in Fig. 3 reaches a value of 260±15 cm^2^ V^−1^ s^−1^ at a temperature of 290 K, which is one to two orders of magnitude higher than for arrays of QDs coupled by surface ligand molecules^12^,^13^,^14^,^15^,^16^, or atomic necks with thickness much less than the QD diameter^7^,^8^ In the present work, the fusion of PbSe QDs via necks with thickness close to the QD diameter has yielded a 2D percolative network with high local mobility as probed in the THz frequency domain. Interestingly, the real THz mobility in the 2D network is only a factor two lower than the highest DC mobility reported for charges moving through PbSe nanowires with a length of microns^31^. The real component of the THz mobility increases somewhat with temperature in contrast to bulk-like band transport as observed for PbSe nanowires^31^,^32^. This suggests that the presence of barriers in the 2D network still limits the efficiency of charge transport, as further discussed below on the basis of DC mobility data.

The barriers experienced by the charge carriers likely correspond to (1) ends of the straight segments in the 2D percolative PbSe network; (2) places at which the necks are thin, leading to smaller electronic coupling between QDs; or (3) energetic difference of a charge at segments with different length. Although the first two factors do not give rise to a temperature dependence of the mobility, the third factor would cause a thermal activation of the mobility. Indeed, the real component of the THz mobility exhibits a slight increase with temperature with the effect being larger at lower frequency. Apparently, at higher temperature charges can more easily move between segments with different energy (length), with the effect being strongest for lower frequency at which a charge moves over a longer distance during the oscillation period of the THz field. Scattering by phonons does not have a strong effect, since that would cause the mobility to decrease with temperature. The distance scale, Δ, probed by the THz field oscillating at radian frequency 
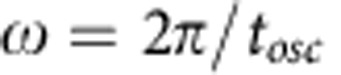
 can be estimated using the Einstein-Smoluchowski relation between real mobility and diffusion coefficient, yielding 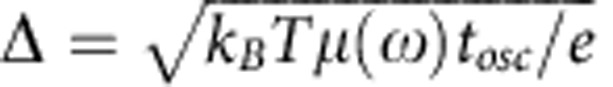. By using this relation with the real mobility data in Fig. 3 for *T*=290 K gives Δ=42 nm at 0.2 THz and Δ=33 nm at 0.6 THz. This implies that a charge moves typically between two straight segments in the percolative network, which have an average length of 20 nm, as discussed above.

**Figure 4:**
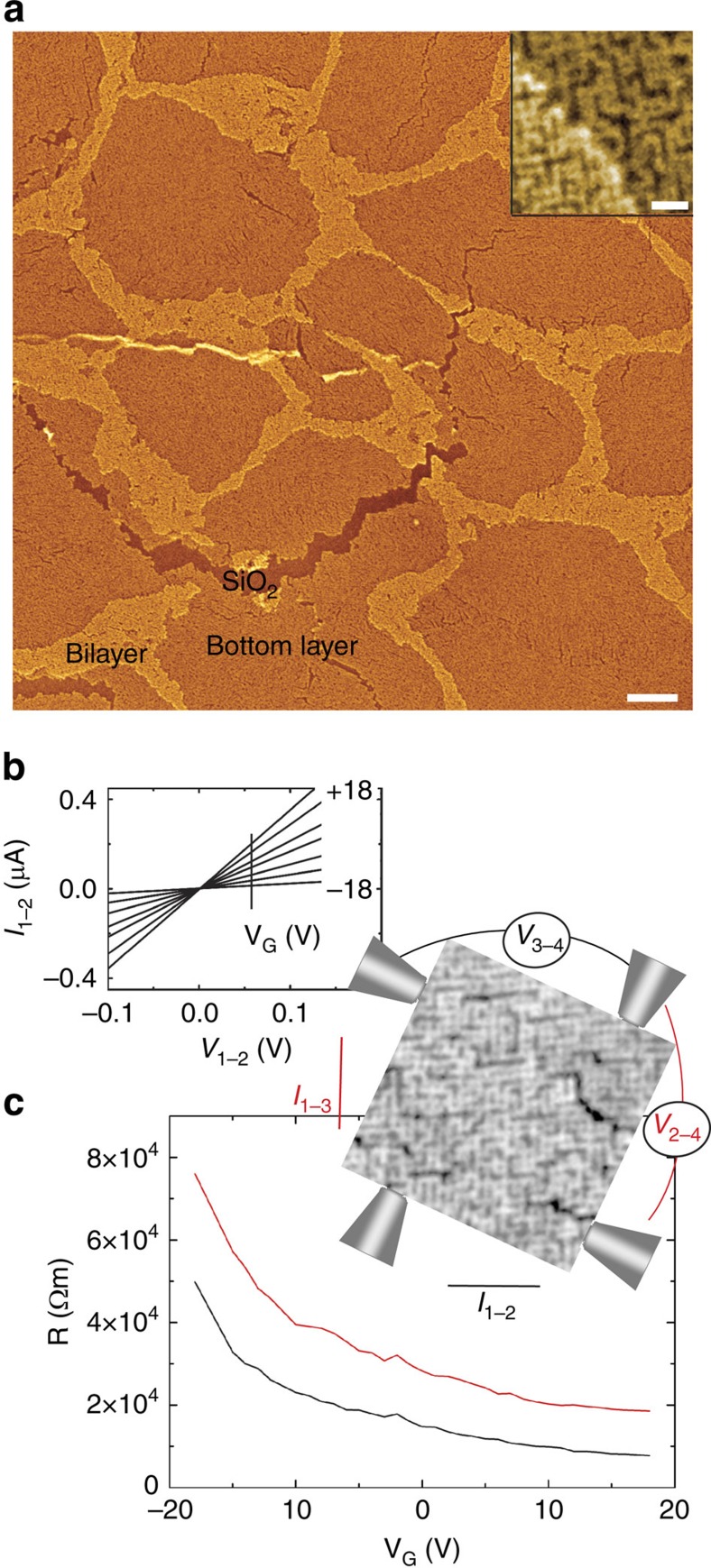
DC transport characteristics. (**a**) SEM image of a network deposited on a 50-nm-thick SiO_2_ layer with the dark alveoli and the bright stripes corresponding to the bottom layer and bilayer, respectively. The scale bar in the lower-right corner represents 200 nm. Inset: high-resolution SEM image obtained at the boundary between a bottom layer and the bilayer. The scale bar in the lower-right corner represents 20 nm. (**b**) Two-terminal current versus voltage at different gate voltages obtained on the bottom layer. (**c**) Four-terminal resistance of a single percolative network measured with a square arrangement of the STM tips at a separation of 500 nm. The direction of the current and the potential drop with respect to the network rows are indicated in the SEM image in the inset.

The DC mobility of charges in the 2D percolative PbSe network was determined from four-point probe electrical measurements with multiple-tip scanning tunnelling microscopy (STM) operated under the guidance of a scanning electron microscope (SEM)^33^. As shown in the SEM image of Fig. 4a, the surface of the sample looks like an alveolar membrane, that can be crossed by narrow tears, revealing the SiO_2_ substrate below. On the basis of high-resolution SEM images (see inset in Fig. 4a), that readily show the 2D percolative networks, the dark alveoli are attributed to the bottom single layer, while the brightest stripes are due to formation of the next layer with limited extent. Although a few elongated alveoli with length of several microns can be found on the surface, allowing a linear arrangement of the four STM tips, the majority of the alveoli are rather rounded with a mean area of 0.24 μm^2^ (see Section 6 of the Supplementary Information). Such an area is appropriate to position the four STM tips with a square arrangement on a single alveolus, with a typical tip separation of 500 nm. Representative back-gated transport characteristics of the probes at room temperature are shown in Fig. 4b,c. The current varies linearly with source-drain bias for the two-tip measurements (Fig. 4b). The symmetry of the current with respect to the origin at positive and negative biases indicates the ohmic nature of the electrical contacts. Note, that the contact resistance was in the range of 1 MΩ and did not significantly change with the tip radius, due to the soft method of making the contacts^33^. The current is further modulated by the gate voltage and exhibits an n-type response, since more positive gate voltages produce an increase in the current. The four-probe resistance as a function of the gate voltage confirms this behaviour (Fig. 4c): the resistance *R* decreases with increasing positive voltages. On driving the current in two different directions, the resistance varies due to the dependence of disorder on direction. Indeed, the resistance is higher when the current is driven perpendicular to the missing rows of the percolative network, visible as black areas in the inset of Fig. 4c.

As the bottom layer contains regions with an additional layer on top of the network, the measured sheet resistance *R*_S_ in an alveoli slightly deviates from the sheet resistance (2π/ln2)*R* of an infinite 2D sheet obtained with four-point square arrangement. Assuming a circular shape for the alveoli, the square four-point probe array in a disk surrounded by a bilayer with a sheet resistance that can be considered as half the value of *R*_S_, gives *R*_S_=(2π/(1+*η*)ln2)*R*, where η is the correction factor ranging between 0.1 and 0.7 depending on the ratio between the fixed tip separation and the radius of the alveoli^34^. For the plot of the resistance shown in Fig. 4c, at zero gate voltage, the four-point probe measurement yields a resistance of 122 kΩ/sq, when the current is driven in the direction where the rows of the 2D percolative network are disrupted least (parallel to the cracks). In the perpendicular direction, a higher resistance of 231 kΩ/sq is found. The field-effect electron mobility is calculated from the derivative of the inverse of the resistance, instead of the current, to eliminate the resistance effects due to the probes:


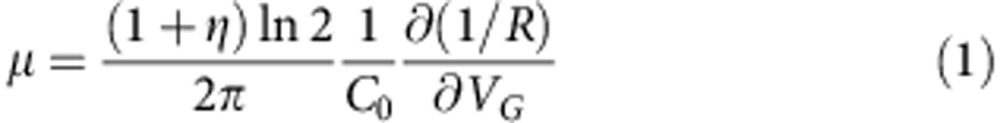


with *C*_0_=6.93 × 10^−8^ Fcm^−2^ the gate capacitance per unit area. The highest mobility is obtained for gate voltages at +10 V. This gives 12 and 3 cm^2^ V^−1^ s^−1^, depending on the direction of electron flow with respect to the disrupted rows in the network. Measurements on different alveoli yielded a mobility of 13±2 cm^2^ V^−1^ s^−1^ for the direction of lowest resistivity.

The THz mobility is more than an order of magnitude higher than the DC mobility, which must be due to barriers limiting the latter on a distance scale less than the STM tip separation (500 nm). Interestingly, the THz mobilities for the 2D percolative networks are two orders of magnitude higher than microwave mobilities found previously for PbSe QDs connected by much thinner necks^8^. Hence, connecting PbSe QDs by necks with thickness close to the QD diameter improves the AC mobility enormously.

The electron density can be estimated from the inverse of the product of the elementary charge, the mobility and the sheet resistance. An electron density of (5±2) × 10^12^ cm^−2^ is obtained, which is comparable to twice the nanocrystal density before the sample is turned into a 2D percolative network. Although the n-type doping of the network can result from an excess of Pb-moieties and ligands through the non-bonding facets, we also speculate that electron beam irradiation might induce the formation of positively charged defects at the surface of the network, giving rise to such a high electron density^35^.

### Theoretical discussion of charge mobility

The observed increase of the mobility with frequency rules out a description of charge motion in terms of the Drude model for isotropic charge scattering, since that would give a real mobility that decreases with frequency according to^26^,^30^.


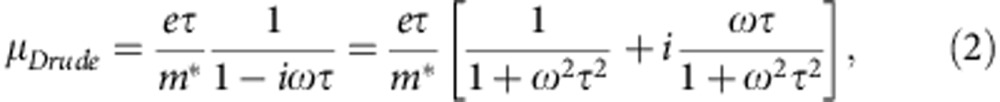


with, m* the effective mass of the charge carrier, τ the average time between scattering events and *i*^2^=−1. In addition, according to the sign convention used in the present work (see Methods), the negative sign of the measured imaginary mobility (see Figs 2c and 3) disagrees with the positive sign of the imaginary Drude mobility in Equation 2.

The increase of the real mobility with frequency is typical for back-scattering at barriers. Effects of such anisotropic scattering are included in the phenomenological model by Smith^36^, which yields the Drude-Smith mobility.


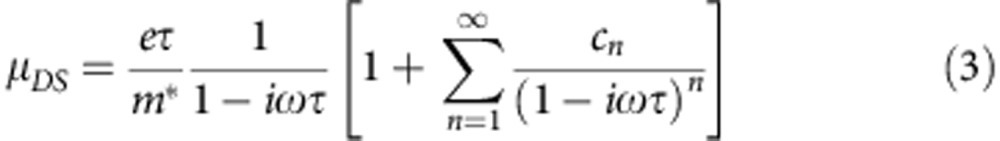


For elastic scattering at angles θ the coefficients *c*_*n*_ are the expectation value 
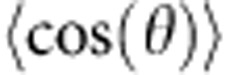
, which is the fraction of the original charge velocity that is retained after *n* scattering events, with negative coefficients resulting from preferential back-scattering^25^,^26^,^36^,^37^. A fit of Equation 3 to the measured mobility data at 290 K in Fig. 3 yields m*=(0.18±0.03)*m*_0_, (*m*_0_ is the free electron mass), *τ*=80±10 fs, *c*_1_=−0.82±0.01 and *c*_*n*≥2_=0. The effective mass and scattering time were found to be independent of temperature, while the back-scattering parameter decreased to −0.86±0.01 at 15 K. The effective mass found in this way is higher than those of electrons and holes in bulk PbSe, which are in the range 0.034–0.07 (ref. 18). This will at least in part be due to the fact the THz mobilities can be somewhat lower than the actual values, as already mentioned above and discussed in Methods and Section 3 of the Supplementary Information. In addition, necks in the 2D percolative cause modification of the band structure compared with the homogeneous crystalline bulk material. Indeed, the effective mass of 0.12*m*_0_ obtained from atomistic tight-binding calculations (see Section 4 of the Supplementary Information) is higher than the value for bulk in agreement with the result from the fit. The negative value of *c*_1_ close to −1 is a signature of significant back-scattering. This can be understood since the limited length of the straight segments in the percolative network do not allow the charge to continue its motion in the forward direction. The finding that the coefficients *c*_*n*≥2_ are zero, implies that the velocity randomizes at the second scattering event.

For the parameter values obtained from the fit to the measured THz mobilities in Fig. 3, the DC mobility is estimated to be 140±15 cm^2^ V^−1^ s^−1^. This is an order of magnitude higher than the experimental DC mobility from the four-probe transport measurements. The difference is not surprising, since the parameters obtained from fitting the Drude-Smith model to the THz mobility are not much influenced by bottlenecks to charge transport on a longer length scale corresponding to the STM tip separation of 500 nm. Nevertheless, we observe THz mobilities that are only a factor of four smaller than the Hall mobility measured with macroscopic PbSe crystals^17^,^18^,^19^.

The Drude-Smith model does not take into account the THz electric field-induced coupling of the wave function of the charge carrier with other electronic states that are coherently delocalized over a segment in the PbSe network. Such coupling leads to polarization of a charge carrier within a segment. The expectation value of the position, *z*, of the charge within a segment is given by *z*=*αE*/*e*, with the α the polarizability and *E* the electric field.

As discussed above, phonon scattering does not significantly affect the mobility of charges in the 2D percolative network of PbSe. If the mean free path for charge scattering by phonons exceeds the typical length of a straight segment in the 2D percolative network, a charge can to a good approximation be considered as coherently delocalized in a segment. The mean free path for scattering by phonons in bulk PbSe is estimated to increase from 9 nm at room temperature to 40 nm at 15 K (see Supplementary Information, Section 5). These distances are comparable to, or longer than, the average segments length (20 nm) in the 2D percolative network, see Fig. 1c. The DC mobility and real component of the THz mobility are then predominantly due to charge motion between segments, while the imaginary mobility is determined by the polarizability of a charge within a single segment. The finding that the polarizability (or imaginary mobility) is virtually independent of temperature (Fig. 3) agrees with the absence of significant scattering of charges by phonons within a straight segment of the percolative network.

The imaginary mobility is related to the polarizability, α, of a charge according μ_*I*_=*αω*/*e*, with ω the radian frequency of the THz field. The close to linear increase of the measured imaginary mobility with frequency in Fig. 3 agrees with this relation. Assuming that a charge on a straight segment of the percolative network behaves like a charge in a 1D box with length *L*, yields 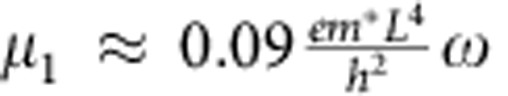, see Supplementary Information Section 5. The slope of the imaginary mobility data in Fig. 3 and m*=0.12 *m*_0_ then gives a segment length *L*=32 nm. This value does not differ much from the 20 nm length of the straight segments estimated from TEM images as in Fig. 1c. The charge transfer frequency, *f*, between segments estimated from the measured DC mobility (13±2 cm^2^ V^−1^ s^−1^), using 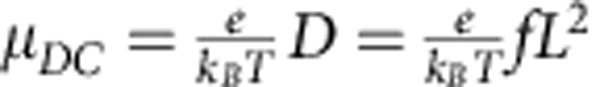, is found to be *f=*4 × 10^10^ s^−1^ at *T*=290 K. Note, that this value is determined mostly by the slowest charge transfer steps in the percolative PbSe network located between the STM tips. The charge transfer frequency estimated above implies that a charge moves at least over a distance of *τ*_*trap*_ *f*=3 segments (100 nm) before being trapped.

## Conclusions

We have studied charge transport in 2D percolative PbSe networks obtained by thermally induced fusion of QDs. Photoexcitation results in formation of free charge carriers rather than neutral excitons. The charge carriers decay by trapping at defects. The mobility of charge carriers does not depend much on temperature and is limited by back-scattering at ends of straight paths in the percolative network. The measured DC mobility is 13±2 cm^2^ V^−1^ s^−1^, while mobilities up to 260±15 cm^2^ V^−1^ s^−1^ were found from THz conductivity measurements. From theoretical analysis of the data, it is inferred that charges are coherently delocalized along segments with a length near 30 nm, and that charge transport occurs by incoherent charge transfer steps between segments. The DC mobility is still limited by the absence of long-range order in the percolative PbSe networks. However, the high THz mobilities are of great promise for eventual application of these networks in optoelectronics and thermoelectrics.

## Methods

### QD synthesis and preparation of 2D PbSe networks

Samples were prepared and stored in nitrogen atmosphere and kept under vacuum during structural and optoelectronic characterization experiments.

PbSe QDs were synthesized following the method of Steckel *et al*.^38^. A mixture of 1.58 g lead acetate trihydrate (99,999%, Sigma-Aldrich), 3.42 g oleic acid (90%, Sigma-Aldrich) and 13.14 g 1-octadecene (90%, Sigma-Aldrich) were heated at 120 °C for 2 h under vacuum. A second mixture containing 1.12 g selenium (99.999%, Alfa Aesar), 0.13 ml diphenylphospine (98%, Sigma-Aldrich) and 14.87 ml trioctylphosphine (90%, Fluka) was prepared by dissolving the Se. Subsequently, the lead-containing solution was heated to 180 °C under nitrogen and the selenium solution was injected. The reaction was allowed to proceed for 40 s at 150 °C after which the reaction was quenched using 15 ml butanol (99.8% anhydrous, Sigma-Aldrich). The crude synthesis mixtures were washed twice by precipitating with methanol, centrifugation and redispersion of the sediment in toluene. This resulted in PbSe QDs with a diameter of 5.8±0.4 nm, as inferred from TEM images.

The oriented attachment of the PbSe QDs to form square 2D superlattices was performed using the method described before^5^,^6^. A volume of 350 μl of a PbSe QD dispersion in toluene at a concentration of 1.75 × 10^−7^ M was placed on top of a volume of 6.5 ml EG (99.8% anhydrous, Sigma-Aldrich) containing 20 nl oleic acid (90%, Sigma-Aldrich) in a vial (diameter 28 mm). The solution is left at 25 °C for 1 h after which the toluene has evaporated and the initial (pseudohexagonal) monolayer is formed (see Supplementary Fig. 1). After heating to 30 °C for 15 min a square superlattice of relatively weakly coupled QDs is formed. Further heating at 50 °C for 15 min and 80 °C for 15 min leads to fusion of QDs and formation of a 2D percolative network with necks of 4.0±0.4 nm. Analysis of TEM images yields a density of PbSe in the network corresponding to 2.4 × 10^12^ QDs per cm^2^.

To enhance the absorbed pump laser fluence and consequently the signal-to-noise ratio in the spectroscopic experiments, we prepared thin films of six-stacked network layers on a quartz substrate. Charge transport from one network layer to another is prevented by the bulky oleic acid surface ligands between the layers. Indeed, the higher-order recombination kinetics of the THz conductivity at the higher photoexcitation fluence are similar for a single layer and a stack of six layers, see Supplementary Fig. 3. If charge transport between layers would be significant, the decay kinetics in the stack of 2D networks would be faster than for a single layer, in contrast to the data.

### TEM characterization

TEM images and electron diffractograms were obtained using a Philips CM30T microscope operating at 200 kV.

### Optical characterization

Optical absorption spectra of QDs in toluene dispersion and thin films of 2D PbSe networks were obtained with a PerkinElmer Lambda 900 spectrometer equipped with an integrating sphere.

### Transient optical absorption measurements

The samples were excited and monitored by pump and probe pulses from a chirped-pulse amplified laser system (Mira-Libra, Coherent Inc.), which runs at 1.4 kHz and delivers pulses of 60 fs at 800 nm. Tuneable infrared and visible pulses (<100 fs) were generated by optical parametric amplification seeded by white light (Topas-800-fs). Pump and probe beams overlapped under a small angle (3°), and were imaged onto InGaAs Pin-photodiodes (Hamamatsu G5853-23, G8605-23). The two beams were spatially separated downstream the sample. Orthogonal polarization of the beams allowed further separation by means of a polarizer. The optical bleach at the band gap 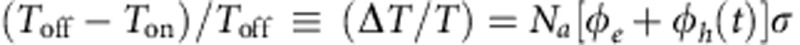 with *T*_off_(*T*_on_) the transmitted probe fluence with pump laser off (on), *N*_*a*_ the number of absorbed pump photons per unit area, *ϕ*_*e*_(*t*)(*ϕ*_*h*_(*t*)) the quantum yield of electrons (holes) at time *t* with respect to the pump pulse, and *σ* the optical bleach cross section of an electron or hole at the band edge. The quantum yield is the number of charge carriers per absorbed pump laser photon.

For temperature-dependent measurements the sample was placed inside a Janis Research helium-closed cycle refrigerator system, which was also used for THz conductivity measurements.

### Terahertz conductivity measurements

The mobility of charge carriers was determined from time-domain THz spectroscopy with the same laser system as used for the transient absorption measurements, as described previously^39^. The sample is excited with 60 fs laser pulses and the photoconductivity due to charges and/or excitons is probed by measuring the change of the amplitude and phase of the THz electric field waveform. This yields the conductivity Δ*σ*(*ω*,*t*) as a function of the frequency, *f*=*ω*/2*π*, of the probing THz field at varying time *t* after sample excitation. The real part of the conductivity is due charge velocity in-phase with a monochromatic driving field, while the imaginary part is due to phase retardation in the motion of charges or due to the polarizability of excitons. THz conductivity measurements on charge carriers provide the frequency dependence of the mobility for motion in an oscillating electric field 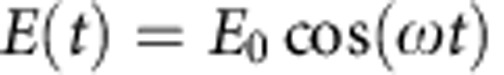. The mobility determines the charge velocity according to 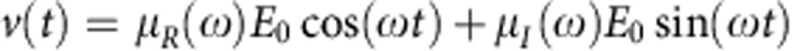, with *μ*_*R*_(*ω*) and *μ*_*I*_(*ω*) the real and imaginary components of the mobility, respectively.

In a home-built set-up a single-cycle THz pulse is generated in LiNbO_3_ and detected in ZnTe. The THz waveform is spectrally encoded using a chirped probe pulse, allowing for single shot THz detection. We measure the differential transmission of the THz waveform 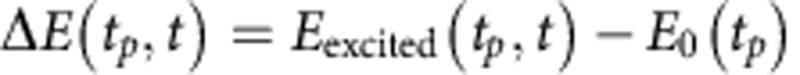, with 
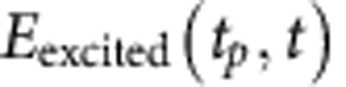
 and 
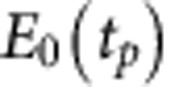
 the transmitted THz electric field in the presence and absence of pumping, respectively, *t*_*p*_ the time between generation and detection of the THz field and *t* the delay between the pump pulse (which excites the sample) and the THz probe pulse. The differential transmission is normalized on *E*_max_, the maximum amplitude of the transmitted THz field in absence of pumping. A Fourier transform with respect to the THz time delay *t*_*p*_, with the convention 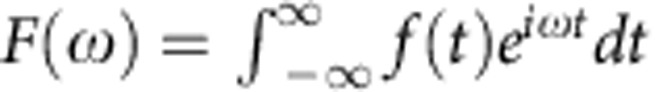, allows retrieval of the complex, frequency-dependent conductivity Δ*σ*(*ω*,*t*) averaged over the thickness *L* of the photoexcited sample:


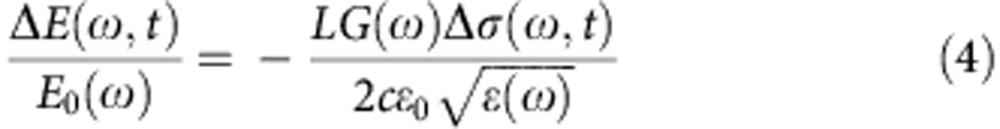


where ω is the Fourier transform with respect to the THz time delay *t*_*p*_, *c* the speed of light, *ɛ*_*0*_ the vacuum permittivity and *ɛ*(*ω*) the dielectric function of the sample. The effective medium parameter 
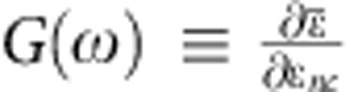
 is the functional derivative of the dielectric constant of the sample (PbSe network and voids) to the dielectric constant *ɛ*_*nc*_ of the mere PbSe network. As discussed in Section 3 of the Supplementary Information 0.4≤*G*(*ω*)≤1. Since this factor cannot be accurately determined for the complex percolative PbSe networks of the present study, it is set equal to one, which results in a mobility that is at most a factor 2.5 lower than the actual value. The dielectric function, *ɛ*(*ω*), was calculated using Bruggeman's theory for inhomogeneous materials^25^,^26^,^37^, with the frequency-dependent dielectric constant of bulk PbSe taken from ref. 40, the medium dielectric constant equal to that of vacuum and a PbSe fill fraction *f*=0.6, as inferred from TEM images of the percolative PbSe network. For the sign of the argument of the exponential used in the Fourier transformation, Drude-like charge transport measured, for example, GaAs (data not shown) yields a positive imaginary component of the mobility. The sum of the product of the quantum yield of electrons and holes and their mobility is obtained using the relation:


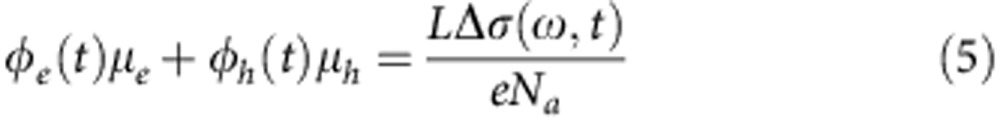


with *N*_*a*_ the number of absorbed pump photons per unit area.

### SEM and four-probe STM current measurements

To carry out SEM and STM measurements on the PbSe superlattices, we deposited PbSe networks onto a degenerately doped Si wafer with a 50-nm-thick thermally grown SiO_2_ layer in a glove box under nitrogen atmosphere. The samples were transferred in a vacuum vessel and then loaded into an ultra-high vacuum system equipped with a four-probe STM and a SEM. Before the electrical measurements, the tungsten tips were thoroughly cleaned by direct resistive heating and their radius was controlled in field emission in the preparation chamber. Such a treatment ensured the formation of reproducible ohmic contacts between the 2D percolative network and the STM tips. Although the approach of the first STM tip to put it in contact with the network was carefully monitored from faint variations of the SEM contrast, the next three STM tips were approached to the network by controlling the tunnelling current that was driven into the network already in contact with the first tip. Establishing contacts in that way allowed to preserve the lattice integrity.

## Additional information

**How to cite this article:** Evers, W. H. *et al*. High charge mobility in two-dimensional percolative networks of PbSe quantum dots connected by atomic bonds. *Nat. Commun.* 6:8195 doi: 10.1038/ncomms9195 (2015).

## Supplementary Material

Supplementary Information Supplementary Figures 1-9, Supplementary Notes 1-2 and Supplementary References.

## References

[b1] Talapin D. V., Lee J.-S., Kovalenko M. V. & Shevchenko E. V. Prospects of colloidal nanocrystals for electronic and optoelectronic applications. Chem. Rev. 110, 389–458 (2010). 19958036 10.1021/cr900137k

[b2] Krahne R. . Physical properties of elongated inorganic nanoparticles. Phys. Rep. 501, 75–221 (2011).

[b3] Bouet C. . Flat colloidal semiconductor nanoplatelets. Chem. Mater. 25, 1262–1271 (2013).

[b4] Schliehe C. . Ultrathin PbS sheets by two-dimensional oriented attachment. Science 329, 550–553 (2010). 20671184 10.1126/science.1188035

[b5] Evers W. H. . Low-dimensional semiconductor superlattices formed by geometric control over nanocrystal attachment. Nano Lett. 13, 2317–2323 (2013). 23050516 10.1021/nl303322k

[b6] Boneschanscher M. P. . Long-range orientation and atomic attachment of nanocrystals in 2D honeycomb superlattices. Science 344, 1377–1380 (2014). 24948734 10.1126/science.1252642

[b7] Baumgardner W. J., Whitham K. & Hanrath T. Confined-but-connected quantum solids via controlled ligand displacement. Nano Lett. 13, 3225–3231 (2013). 23777454 10.1021/nl401298s

[b8] Sandeep C. S. S. . Epitaxially connected PbSe quantum-dot films: controlled neck formation and optoelectronic properties. ACS Nano 8, 11499–11511 (2014). 25347299 10.1021/nn504679k

[b9] Kalesaki E. . Dirac cones, topological edge states, and nontrivial flat bands in two-dimensional semiconductors with a honeycomb nanogeometry. Phys. Rev. X 4, 011010 (2014).

[b10] Kalesaki E., Evers W. H., Allan G., Vanmaekelbergh D. & Delerue C. Electronic structure of atomically coherent square semiconductor superlattices with dimensionality below two. Phys. Rev. B 88, 115431 (2013).

[b11] Delerue C. From semiconductor nanocrystals to artificial solids with dimensionality below two. Phys. Chem. Chem. Phys. 16, 25734–25740 (2014). 25045765 10.1039/c4cp01878h

[b12] Talgorn E. . Unity quantum yield of photogenerated charges and band-like transport in quantum-dot solids. Nat. Nanotechnol. 6, 733–739 (2011). 21946709 10.1038/nnano.2011.159

[b13] Liu Y. . PbSe quantum dot field-effect transistors with air-stable electron mobilities above 7 cm^2^ V^−1^s^−1^. Nano Lett. 13, 1578–1587 (2013). 23452235 10.1021/nl304753n

[b14] Oh S. J. . Designing high-performance PbS and PbSe nanocrystal electronic devices through stepwise, post-synthesis, colloidal atomic layer deposition. Nano Lett. 14, 1559–1566 (2014). 24499242 10.1021/nl404818z

[b15] Jang J., Liu W., Son J. S. & Talapin D. V. Temperature-dependent Hall and field-effect mobility in strongly coupled all-inorganic nanocrystal arrays. Nano Lett. 14, 653–662 (2014). 24467484 10.1021/nl403889u

[b16] Guglietta G. W. . Lifetime, mobility, and diffusion of photoexcited carriers in ligand-exchanged lead selenide nanocrystal films measured by time-resolved terahertz spectroscopy. ACS Nano 9, 1820–1828 (2015). 25644854 10.1021/nn506724h

[b17] Allgaier R. S. & Scanlon W. W. Mobility of electrons and holes in PbS, PbSe, and PbTe between room temperature and 4.2 degrees K. Phys. Rev. 111, 1029–1037 (1958).

[b18] Dalven R. A review of semiconductor properties of PbTe, PbSe, PbS and PbO. Infrared Phys. 9, 141–184 (1969).

[b19] Schlicht U. & Gobrecht K. H. The mobility of free carriers in PbSe crystals. J. Phys. Chem. Solids 34, 753–758 (1973).

[b20] Wolcott A. . Anomalously large polarization effect responsible for excitonic red shifts in PbSe quantum dot solids. J. Phys. Chem. Lett. 2, 795–800 (2011).

[b21] Dollefeld H., Weller H. & Eychmuller A. Particle-particle interactions in semiconductor nanocrystal assemblies. Nano Lett. 1, 267–269 (2001).

[b22] Murphy J. E., Beard M. C. & Nozik A. J. Time-resolved photoconductivity of PbSe nanocrystal arrays. J. Phys. Chem. B 110, 25455–254461 (2006). 17165993 10.1021/jp0646123

[b23] Wehrenberg B. L., Wang C. J. & Guyot-Sionnest P. Interband and intraband optical studies of PbSe colloidal quantum dots. J. Phys. Chem. B 106, 10634–10640 (2002).

[b24] Kigel A., Brumer M., Maikov G. I., Sashchiuk A. & Lifshitz E. Thermally activated photoluminescence in lead selenide colloidal quantum dots. Small 5, 1675–1681 (2009). 19347855 10.1002/smll.200801378

[b25] Nemec H., Kuzel P. & Sundstrom V. Charge transport in nanostructured materials for solar energy conversion studied by time-resolved terahertz spectroscopy. J. Photochem. Photobiol. A Chem. 215, 123–139 (2010).

[b26] Ulbricht R., Hendry E., Shan J., Heinz T. F. & Bonn M. Carrier dynamics in semiconductors studied with time-resolved terahertz spectroscopy. Rev. Mod. Phys. 83, 543–586 (2011).

[b27] Cunningham P. D. Accessing terahertz complex conductivity dynamics in the time-domain. IEEE Trans. Terahertz Sci. Technol. 3, 494–498 (2013).

[b28] Johnston D. C. Stretched exponential relaxation arising from a continuous sum of exponential decays. Phys. Rev. B 74, 184430 (2006).

[b29] Hilt O. & Siebbeles L. D. A. Time and frequency dependent charge carrier mobility of one-dimensional chains with energetic disorder. Chem. Phys. Lett. 269, 257–262 (1997).

[b30] Grozema F. C. & Siebbeles L. D. A. Mechanism of charge transport in self-organizing organic materials. Int. Rev. Phys. Chem. 27, 87–138 (2008).

[b31] Graham R. & Yu D. High carrier mobility in single ultrathin colloidal lead selenide nanowire field effect transistors. Nano Lett. 12, 4360–4365 (2012). 22823181 10.1021/nl302161n

[b32] Oh S. J., Kim D. K. & Kagan C. R. Remote doping and Schottky barrier formation in strongly quantum confined single PbSe nanowire field-effect transistors. ACS Nano 6, 4328–4334 (2012). 22512336 10.1021/nn3009382

[b33] Berthe M. . in Atomic Scale Interconnection Machines ed Joachim C. Springer (2012).

[b34] Swartzendruber L. J. Four-point probe measurement of non-uniformities in semiconductor sheet resistivity. Solid State Electron. 7, 413–422 (1964).

[b35] Durand C. . Persistent enhancement of the carrier density in electron irradiated InAs nanowires. Nanotechnology 24, 275706 (2013). 23764855 10.1088/0957-4484/24/27/275706

[b36] Smith N. V. Classical generalization of the Drude formula for the optical conductivity. Phys. Rev. B 64, 155106 (2001).

[b37] Lloyd-Hughes J. & Jeon T.-I. A Review of the terahertz conductivity of bulk and nano-materials. J. Infrared Millimeter Terahertz Waves 33, 871–925 (2012).

[b38] Steckel J. S., Yen B. K. H., Oertel D. C. & Bawendi M. G. On the mechanism of lead chalcogenide nanocrystal formation. J. Am. Chem. Soc. 128, 13032–13033 (2006). 17017765 10.1021/ja062626g

[b39] Kunneman L. T. . Nature and decay pathways of photoexcited states in CdSe and CdSe/CdS nanoplatelets. Nano Lett. 14, 7039–7045 (2014). 25366327 10.1021/nl503406a

[b40] Hyun B.-R. . Far-infrared absorption of PbSe nanorods. Nano Lett. 11, 2786–2790 (2011). 21627094 10.1021/nl201115h

