# Supplementary material for: High charge mobility in two-dimensional percolative networks of PbSe quantum dots connected by atomic bonds
Source: Nat Commun. 2015 Sep 24;6:8195. doi: 10.1038/ncomms9195 (PMC4598357; doi:10.1038/ncomms9195)
Supplement: Supplementary Information — Supplementary Figures 1-9, Supplementary Notes 1-2 and Supplementary References. [file ncomms9195-s1.pdf]

Supplementary figures

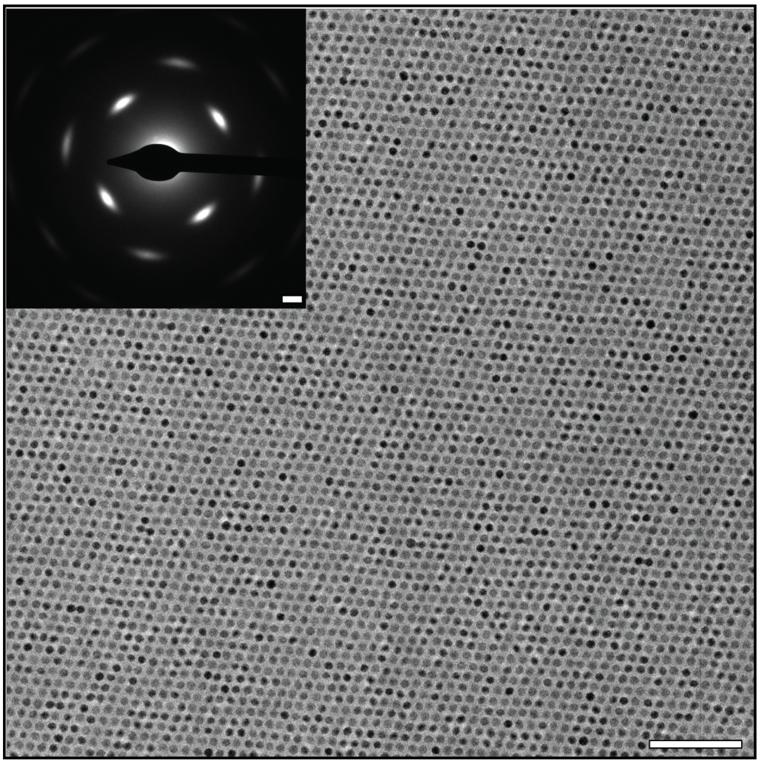

Supplementary Figure 1. TEM and electrodiffractrogram of pseudo-hexagonally packed PbSe QDs. Layer of pseudo-hexagonally packed PbSe QDs obtained after evaporation of the toluene solvent, which forms the preface to the two-dimensional superstructure with square geometry. From the appearance of spots rather than rings in the electrodiffractogram in the inset it is evident that the crystal planes in all QDs have the same orientation. The scale bars represent 60 nm and 1 nm^{-1} for the TEM image and electrodiffractogram, respectively

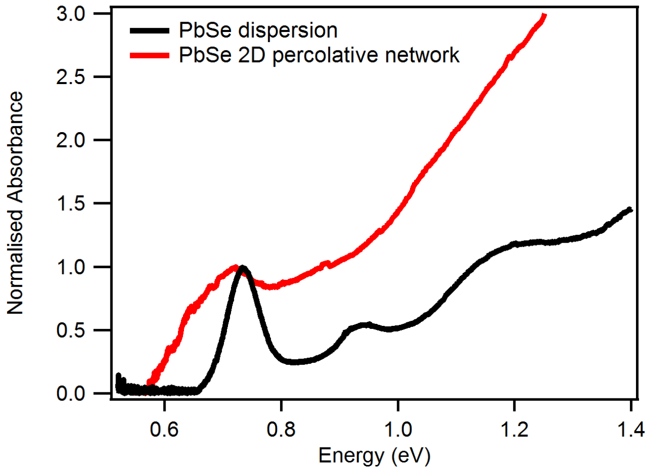

Supplementary Figure 2. Optical absorption spectra of PbSe dispersion and 2D percolative network. Optical absorption spectrum of the film of six layers of 2D percolative PbSe networks (red) studied in the present work, together with the spectrum of a dispersion of the PbSe QDs from which the networks were produced (black).

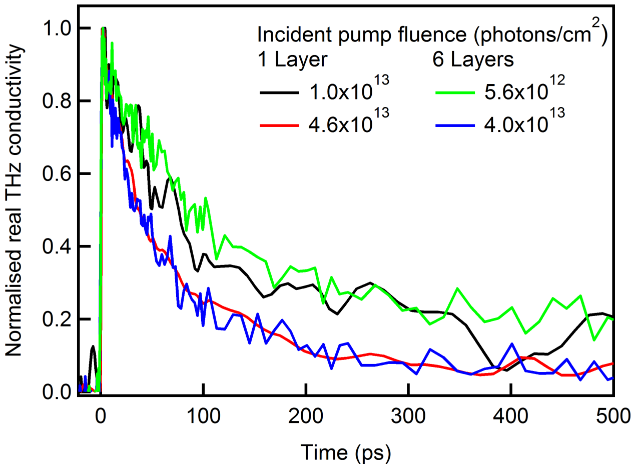

Supplementary Figure 3. Normalised terahertz conductivity at various fluences for the 1 and 6 layered samples. Decay kinetics of the real component of the THz conductivity for films with one or six 2D percolative network layers and laser pump fluences as indicated. The decay kinetics do not depend on the number of layers, which implies that charge transport between layers is negligible.

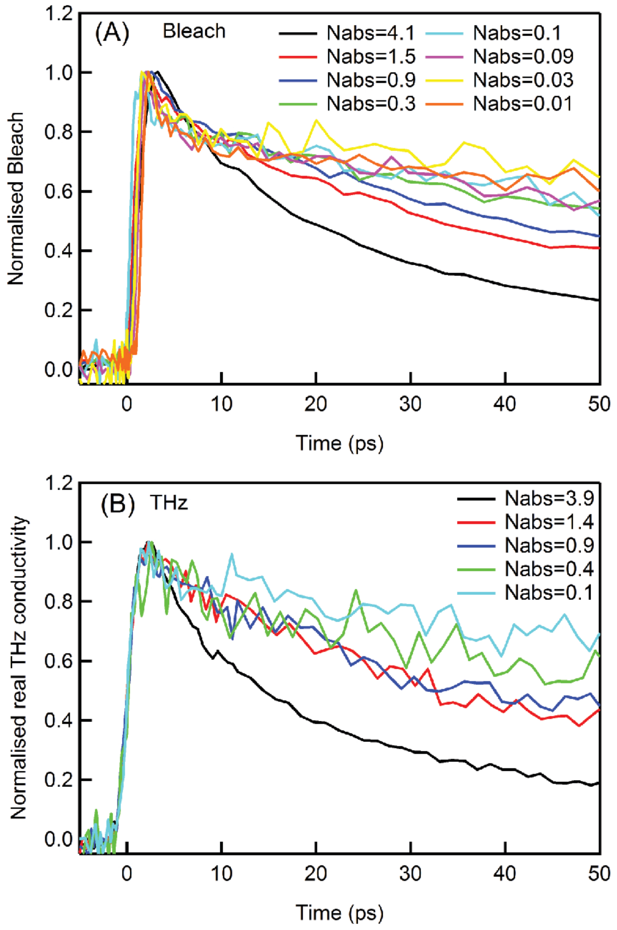

Supplementary Figure 4. Effect of absorbed laser pump fluence on decay kinetics of optical bleach and THz conductivity. The decay kinetics is independent of pump fluence if the number of excitations per QD volume (Nabs) is less than 0.4.

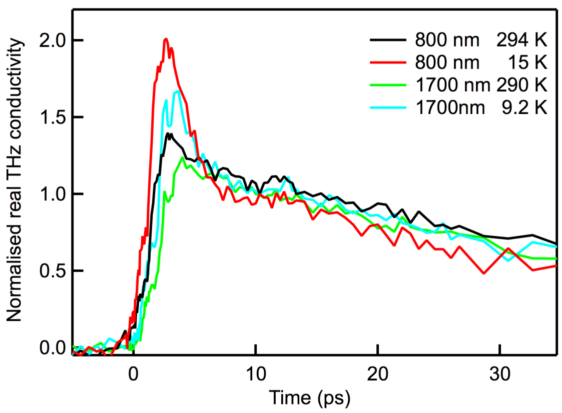

Supplementary Figure 5. THz conductivity for photoexcitation at 800 and 1700 nm at temperatures as indicated. The amplitudes of the transients were normalised at 10 ps.

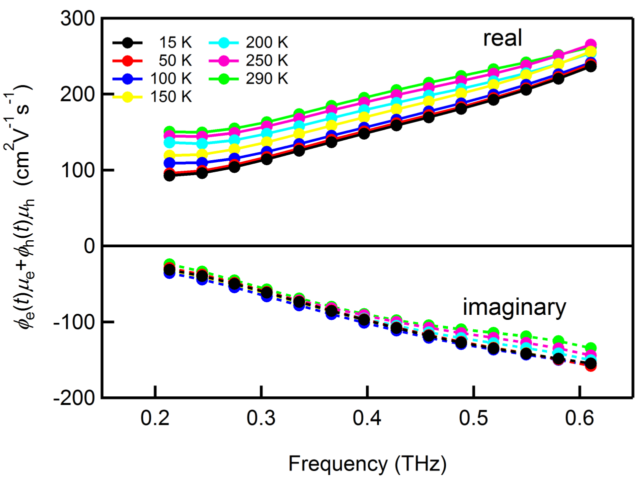

Supplementary Figure 6. Frequency dependence of the mobility of the long-lived charge carriers. Real (upper panel) and imaginary (lower panel) components of the charge mobility at temperatures as indicated, averaged over pump-probe time delays in the interval 8-12 ps.

In the section "Photogeneration and decay of charge carriers" of the main article it is mentioned that the THz conductivity can only be due to free charges. The assignment of the THz conductivity to free charges, rather than excitons or plasmons, is corroborated below. The frequency dependence of the real and imaginary mobility is shown in Fig. 3 of the main text and in Fig. S6. From the experimental data one can deduce the ratio of imaginary to real mobility; i.e.

$$R(\omega) \equiv \left| \frac{\mu_{im}(\omega)}{\mu_{re}(\omega)} \right|, \quad (1)$$

which is shown in Fig. S7 for $T = 290$ K (data at lower temperatures are similar, see Fig. S6). For excitons or plasmons this ratio is given by[1,2]

$$R_{\begin{cases} plasmon \\ exciton \end{cases}}(\omega) = \left| \frac{\omega_{res}^2 - \omega^2}{\omega\gamma} \right| \sim \left| \frac{\omega_{res}^2}{\omega} - \omega \right|. \quad (2)$$

with ω_{res} the radian resonance frequency and γ the width of the resonance. The latter only scales the ratio $R(\omega)$ and does not affect its frequency dependence. Since the measured real component of the THz conductivity in Fig. 3 increases with frequency, a possible resonance due to excitons or plasmons must occur above the highest experimental frequency of 0.6 THz. Therefore in Fig. S7 two different model curves for $R_{\begin{cases}plasmon\\exciton\end{cases}}(\omega)$ are shown in red for the lowest possible resonant frequency of 0.6 (continuous) and a higher frequency of 2 THz (dashed). The decrease of the model curves for excitons and plasmons is in clear contrast to the measured increase of the ratio. Hence, the measured THz conductivity cannot be due to excitons or plasmons and must result from free charges.

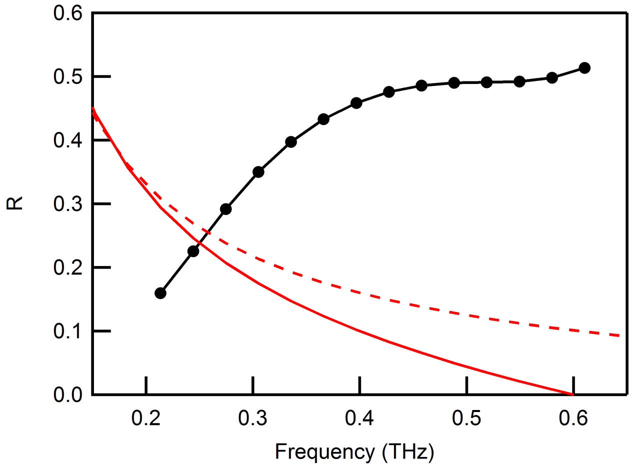

Supplementary Figure 7. Ratio of the imaginary and real mobility. Experimental results obtained from the data at $T = 290$ K in Fig. 3 (black markers) together with model curves for excitons or plasmons with resonance frequencies of 0.6 (continuous red curve) and 2 THz (dashed red curve).

As described in the Methods section, the relative change in the THz electric field is related to the photoconductivity according to

$$\frac{\Delta E(\omega,t)}{E_0(\omega)} = -\frac{LG(\omega)\Delta\sigma(\omega,t)}{2c\varepsilon_0\sqrt{\varepsilon(\omega)}} \tag{3}$$

Below it is argued that the effective medium parameter $G(\omega) \equiv \frac{\partial \bar{\varepsilon}}{\partial \varepsilon_{nc}}$ is real-valued with a value between 0.4 and 1. The lower limit of 0.4 is obtained for a three-dimensional distribution of nanocrystals with space filling fraction f = 0.6, as determined from TEM images. According to Bruggeman's theory the dielectric constant $\bar{\varepsilon}$ of the sample depends on ε_{host} of the host (vacuum) and ε_{nc} of the PbSe network as[1]

$$f \frac{\varepsilon_{nc} - \bar{\varepsilon}}{\varepsilon_{nc} + 2\bar{\varepsilon}} = (1-f) \frac{\bar{\varepsilon} - \varepsilon_{host}}{\varepsilon_{host} + 2\bar{\varepsilon}}. \tag{4}$$

It follows from the above that

$$\left.\begin{array}{l} \bar{\varepsilon} = \varepsilon_{host} \dfrac{G+1}{4} H \\ 5 \dfrac{\partial \bar{\varepsilon}}{\partial \varepsilon_{nc}} = G \dfrac{1-4\eta}{H^2 + 8\eta} + 1 + G \end{array}\right\} \quad \left\{\begin{array}{l} H \equiv \dfrac{4\eta + 1}{5} \\ G^2 = 1 + 8 \dfrac{\eta}{H^2} \\ \eta \equiv \dfrac{\varepsilon_{nc}}{\varepsilon_{host}} \end{array}\right. \tag{5}$$

The effective medium parameter $G(\omega) \equiv \frac{\partial \bar{\varepsilon}}{\partial \varepsilon_{nc}}$ is plotted in Fig. S8 as a function of frequency, upon using Hyun's data[3] for the dielectric constant ε_{nc} of PbSe nanocrystals, and ε_{host}=1 for vacuum. It is evident that for the frequencies of interest in the current work ($\leq$ 0.6 THz) the function $G(\omega)$ is real-valued with a value of 0.4, which is a lower limit to that for the PbSe networks studied.

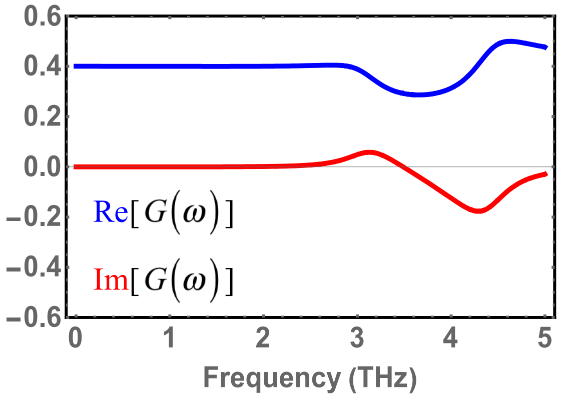

Supplementary Figure 8. Real (blue) and imaginary (red) parts of the effective medium parameter $G(\omega)$**.**

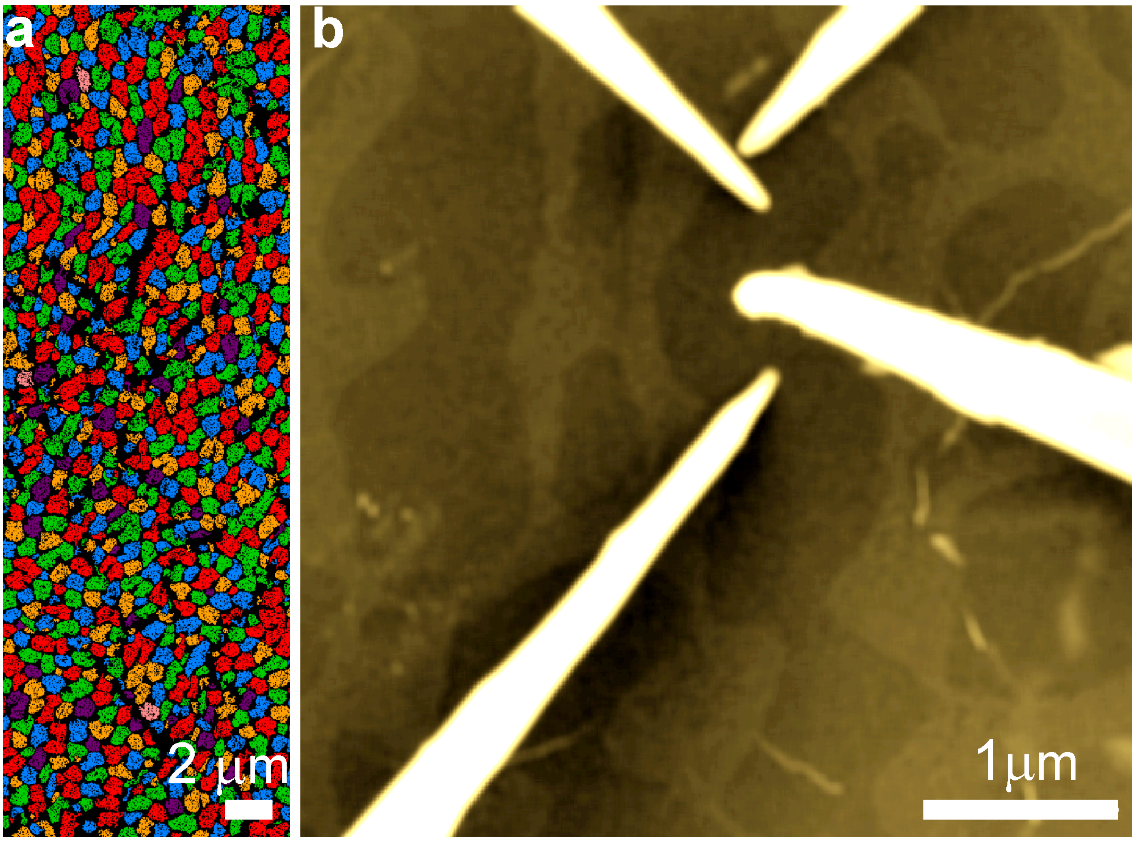

Supplementary Figure 9. Network morphology for the DC measurements. a, SEM image of the 2D percolative PbSe network that allows to perform a mean size analysis of the alveoli. The multicolor map allows to discriminate between contiguous alveoli. b, SEM image of the 2D percolative PbSe network with four STM tips in contact with the bottom single layer in an elongated alveola.

Supplementary note 1

Band structure and effective mass in square 2D networks of quantum dots

In this section we theoretically estimate the effective mass of charges in periodic square 2D networks of PbSe QDs that are fused by necks of diameter *d*. Atomistic tight-binding calculations show that the lowest conduction (highest valence) band is formed by the nearest-neighbour coupling between electron (hole) quantum dot states with S envelope wave-function.[4,5] If we neglect inter-valley couplings that only have minor effects, the band dispersion can be approximated by $E(\mathbf{k}) = E_s + 2t[\cos(k_x a) + \cos(k_y a)]$ where E_s is the on-site energy, a is the centre-to-centre distance between the quantum dots, and t is the hopping integral which can be positive or negative for reasons discussed previously.[4] The width of the band is $8|t|$ and the effective mass at the bottom (top) of the band (**k**=0) is $m^* = \hbar^2/(2|t|a^2)$. The calculations yield similar widths of the lowest conduction and highest valence band.[4,5] The calculated band widths for square networks of QDs with diameter 6.0 nm in Fig. 3 of Ref. [5] can be considered representative for the experimentally studied networks of QDs with diameter of 5.8 ± 0.4 nm. Taking the experimental width of the necks, d = 4.0 nm and the centre-to-centre distance between the QDs of 6.4 nm, we find from the data in Fig. 3 of Ref. [5] that the hopping integral t = 8 meV and that $m^* = 0.12\ m_0$.

Supplementary note 2

Theoretical description of charges coherently delocalized in straight segments of the 2D percolative PbSe network

The mobility of electrons and holes in bulk PbSe is about 10^3 $cm^2V^{-1}s^{-1}$ at room temperature and increases to values near $2x10^4$ $cm^2V^{-1}s^{-1}$ at temperature of 15 K.[6-8] Taking the effective mass 0.05 times the free electron mass,[6-8] yields a scattering time $\tau = \mu m^*/e$ = 30 fs at room temperature and 600 fs at 15 K. With a thermal velocity $v = \sqrt{k_B T/m^*}$ = $3x10^5$ m/s at room

temperature and 7x10^4 m/s at 15 K, the mean free path for scattering on phonons is $\lambda = v\tau$ = 9 nm at room temperature and increases to 42 nm at 15 K.

The polarisability of a charge on a straight segment of length L is described by the 1D particle-in-a-box model. Hence, the wave functions and energies are[9]

$$\Psi_n(z) = \sqrt{\frac{2}{L}}\sin\left(n\pi\frac{z}{L}\right) \tag{6}$$

and

$$E_n = \frac{n^2h^2}{8m^*L^2} \tag{7}$$

with the quantum number n = 1, 2, 3, .. and h Planck's constant. For the average length L = 20 nm of the straight segments in the percolative PbSe network, an effective mass of a charge moving *within* a segment equal to the mean bulk value, $m^* = 0.05\ m_0$, it is found that the spacing between successive energy levels is 56 meV or more. Since the THz photon energies in the experiments (0.8 - 2.4 meV) are small compared to the energy level spacing, the frequency dependence of the polarisability is negligible, in agreement with the close to linear increase of the imaginary mobility with frequency in Fig. 3. The polarisability for a segment parallel to the external electric field is[9]

$$\alpha = 2\sum_{n'=1}^{\infty}\frac{\left|<\Psi_{2n'}|ez|\Psi_{n=1}>\right|^2}{E_{2n'}-E_{n=1}} = 2\left(\frac{8eL}{\pi^2}\right)^2\sum_{n'=1}^{\infty}\frac{\left|\left(2n'\Big/\left(4n'^2-1\right)^2\right)\right|^2}{E_{2n'}-E_{n=1}} =$$

$$= \frac{4096e^2m^*L^4}{\pi^2h^2}\sum_{n'=1}^{\infty}\frac{n'^2}{\left(4n'^2-1\right)^5} \approx 0.17\frac{e^2m^*L^4}{h^2} \quad . \tag{8}$$

In the experiments the segments are aligned randomly with respect to the THz electric field vector. Hence, the expression for the polarisability derived above must be averaged over all angles, ϕ, between the segment and the electric field vector, so that

$$\alpha_{measured} = \frac{\int_0^{2\pi} \cos^2(\phi)\, d\phi}{\int_0^{2\pi} d\phi}\, \alpha = \frac{1}{2}\alpha \approx 0.09 \frac{e^2 m^* L^4}{h^2} \quad (9).$$

The measured imaginary mobility then becomes

$$\mu_{imag} = \frac{\alpha\omega}{e} \approx 0.09 \frac{e m^* L^4}{h^2}\omega \quad . \quad (10)$$

Supplementary References

1 Ulbricht, R., Hendry, E., Shan, J., Heinz, T. F. & Bonn, M. Carrier dynamics in semiconductors studied with time-resolved terahertz spectroscopy. *Rev. Mod. Phys.* **83**, 543-586, doi:10.1103/RevModPhys.83.543 (2011).

2 Lloyd-Hughes, J. & Jeon, T.-I. A Review of the terahertz conductivity of bulk and nano-materials. *J. Infrared Millimeter and Terahertz Waves* **33**, 871-925, doi:10.1007/s10762-012-9905-y (2012).

3 Hyun, B.-R. *et al.* Far-infrared absorption of PbSe nanorods. *Nano Lett.* **11**, 2786 - 2790 (2011).

4 Kalesaki, E., Evers, W. H., Allan, G., Vanmaekelbergh, D. & Delerue, C. Electronic structure of atomically coherent square semiconductor superlattices with dimensionality below two. *Phys Rev B* **88**, 115431, doi:Doi 10.1103/Physrevb.88.115431 (2013).

5 Delerue, C. From semiconductor nanocrystals to artificial solids with dimensionality below two. *Phys. Chem. Chem. Phys.* **16**, 25734-25740, doi:10.1039/c4cp01878h (2014).

6 Allgaier, R. S. & Scanlon, W. W. Mobility of electrons and holes in PbS, PbSe, and PbTe between room temperature and 4.2 degrees K. *Phys. Rev.* **111**, 1029-1037, doi:10.1103/PhysRev.111.1029 (1958).

7 Dalven, R. A review of semiconductor properties of PbTe, PbSe, PbS and PbO. *Infrared Physics* **9**, 141-184, doi:10.1016/0020-0891(69)90022-0 (1969).

8 Schlicht. U & Gobrecht, K. H. Mobility of free carriers in PbSe crystals. *J. Phys. Chem. Solids* **34**, 753-758, doi:10.1016/s0022-3697(73)80183-0 (1973).

9 Atkins, P. & Paula, J. D. *Atkins' Physical Chemistry*. 9th edn, (Oxford University Press, 2010).
